# High endothelial venules are associated with microsatellite instability, hereditary background and immune evasion in colorectal cancer

**DOI:** 10.1038/s41416-019-0514-6

**Published:** 2019-07-30

**Authors:** Pauline L. Pfuderer, Alexej Ballhausen, Florian Seidler, Hans-Jürgen Stark, Niels Grabe, Ian M. Frayling, Ann Ager, Magnus von Knebel Doeberitz, Matthias Kloor, Aysel Ahadova

**Affiliations:** 1 Department of Applied Tumour Biology, Institute of Pathology, University Hospital Heidelberg, Heidelberg, Germany; 2 Clinical Cooperation Unit Applied Tumour Biology, DKFZ, Heidelberg, Germany; 3 Molecular Medicine Partnership Unit (MMPU), University Hospital Heidelberg, Heidelberg, Germany; 4 Hamamatsu Tissue Imaging and Analysis (TIGA) Center, Heidelberg, Germany; 5 Department of Medical Oncology, National Center for Tumour Diseases (NCT), Heidelberg, Germany; 6 Inherited Tumour Syndromes Research Group, Institute of Cancer & Genetics, Cardiff University School of Medicine, Cardiff, UK; 7 Division of Infection and Immunity, School of Medicine and Systems Immunity Research Institute, Cardiff University, Cardiff, UK

**Keywords:** Immunoediting, Immune evasion

## Abstract

**Background:**

Microsatellite-unstable (MSI) tumours show a high load of mutational *neo*antigens, as a consequence of DNA mismatch repair deficiency. Consequently, MSI tumours commonly present with dense immune infiltration and develop immune evasion mechanisms. Whether improved lymphocyte recruitment contributes to the pronounced immune infiltration in MSI tumours is unknown. We analysed the density of high endothelial venules (HEV) and postcapillary blood vessels specialised for lymphocyte trafficking, in MSI colorectal cancers (CRC).

**Methods:**

HEV density was determined by immunohistochemical staining of FFPE tissue sections from MSI (*n* = 48) and microsatellite-stable (MSS, *n* = 35) CRCs. Associations with clinical and pathological variables were analysed.

**Results:**

We found elevated HEV densities in MSI compared with MSS CRCs (median 0.049 vs 0.000 counts/mm^2^, respectively, *p* = 0.0002), with the highest densities in Lynch syndrome MSI CRCs. Dramatically elevated HEV densities were observed in *B2M-*mutant Lynch syndrome CRCs, pointing towards a link between lymphocyte recruitment and immune evasion (median 0.485 vs 0.0885 counts/mm^2^ in *B2M*-wild-type tumours, *p* = 0.0237).

**Conclusions:**

Our findings for the first time indicate a significant contribution of lymphocyte trafficking in immune responses against MSI CRC, particularly in the context of Lynch syndrome. High HEV densities in *B2M*-mutant tumours underline the significance of immunoediting during tumour evolution.

## Background

Genomic instability in cancer can arise through two main mechanisms: chromosomal and microsatellite instability. Microsatellite instability (MSI) is caused by a defect in the DNA mismatch repair (MMR) system, which can occur sporadically by epigenetic alterations in the *MLH1* gene, or in the context of the most common inherited cancer syndrome, named Lynch syndrome, by mutational inactivation of one of the four MMR genes (*MLH1*, *MSH2*, *MSH6* and *PMS2*).^1^ As a consequence, mismatches occurring during DNA replication cannot be corrected, and small insertion/deletion mutations accumulate at short repetitive stretches called microsatellites.

Accumulation of uncorrected mutations at microsatellites leads to shifts of the translational reading frame and generation of long stretches of the so-called frameshift peptides (FSPs), potentially containing highly immunogenic epitopes.^2^ The high immunogenicity of MSI-induced *neo*antigens is thought to explain the dense infiltration of tumour tissue with cytotoxic T cells,^3^ and hence the favourable prognosis of patients with MSI cancers, in contrast to patients with MSS cancers,^4–6^ and the particularly good response to immune checkpoint blockade.^7^ In addition, 30% of MSI CRCs present with mutations of the *B2M* gene encoding for the light chain of MHC class I molecule.^8,9^ Such *B2M* mutations lead to the synthesis of a truncated B2M protein, thereby completely abrogating MHC class I-associated antigen expression and contributing to immune evasion. The outgrowth of *B2M*-mutant tumour cell clones represents a manifestation of cancer immunoediting concept^10–12^ and further supports high immunogenicity of MSI CRCs and their active immune surveillance.^13^ However, the mechanisms of lymphocyte recruitment that may contribute to the pronounced immune infiltration of MSI cancers have not yet been determined.

High endothelial venules (HEVs) are postcapillary venules specialised for lymphocyte trafficking.^14^ Endothelial cells lining HEVs express on their cell surface peripheral node addressins (PNAd), molecules which are important for lymphocyte homing. HEVs attract naive and central memory lymphocytes from the bloodstream into lymph nodes, where they scan antigen-presenting cells for activating or tolerogenic stimuli.^15^ Recently, HEVs were also found in tumours of different origin, including colorectal, breast, ovarian, lung cancer and melanomas,^16,17^ implying the possibility of direct lymphocyte recruitment into cancer tissue. Moreover, the density of HEVs has been shown to correlate with T-cell infiltration in tumours and survival of patients with breast cancer.^16^

In this study, we analysed whether HEVs play a role in the pronounced immune cell infiltration of MSI CRCs by assessing their density in MSI and MSS CRC tissues. Moreover, we aimed to identify additional clinicopathological factors potentially associated with the presence of HEVs in colorectal tumours.

## Methods

### Tumour samples

Formalin-fixed paraffin-embedded (FFPE) tumour tissue blocks were collected at the Department of Applied Tumour Biology, Institute of Pathology, University Hospital Heidelberg, as part of the German HNPCC Consortium. Informed and written consent was provided by all patients, and the study approval was obtained from the Institutional Ethics Committee.

### Immunohistochemical staining

FFPE tissue sections (2 µm) were used for immunohistochemical staining. Deparaffinisation, rehydration and staining were performed according to standard protocols as described previously.^18,19^ For HEV staining, the rat monoclonal antibody MECA-79 (Santa Cruz Biotechnology, Dallas, TX, USA, clone SC-19602) was applied as a primary antibody at 1:750 dilution; as a secondary antibody, the biotinylated goat anti-rat antibody (Vector Laboratories, Burlingame, CA, USA) was used at 1:100 dilution. For PD-L1 staining, anti-PD-L1 rabbit monoclonal antibody (Ventana Medical Systems, Tucson, AZ, USA, clone SP263) was used, CD3 staining was performed using the α-CD3 (Abcam, Cambridge, UK, clone SP7) rabbit monoclonal antibody at 1:200 dilution, PD1 staining was performed using the α-PDCD1 mouse monoclonal antibody (1:50 dilution, Abcam, clone NAT105), CD20 staining was performed using the α-CD20 (Santa Cruz, clone D-10) mouse monoclonal antibody at 1:200 dilution, and cytokeratin staining was performed using mouse monoclonal antibody, clone AE1/AE3 (Dako) at 1:50 dilution; as a secondary antibody in PD-L1, PD1 and CD3 staining, biotinylated horse anti-mouse/anti-rabbit antibody (Vector Laboratories, Burlingame, CA, USA) was used at 1:50 dilution. Staining visualisation was performed using the Vectastain elite ABC detection system (Vector Laboratories, Burlingame, CA, USA) and 3,3′-Diaminobenzidine (Dako North America Inc., Carpinteria, CA, USA) as a chromogen. Finally, slides were counterstained with haematoxylin.

### Evaluation of HEV density

The HEVs were identified through positive staining with MECA-79 antibody and histomorphological properties characteristic of cells comprising HEVs, such as a cuboidal shape of high endothelial cells.^15,17^ Spatially separated MECA-79-positive HEVs were counted under a light microscope at ×4 objective magnification. Representative MECA-79-positive HEVs are shown in Supplementary Fig. S1. A randomly selected subset of slides was evaluated by a second observer (*r* = 0.93, *p* = 0.00012, Spearman correlation). The observers were blinded for any clinical parameters, such as age, gender, stage, hereditary background, and molecular tumour characteristics. All counts were normalised per mm^2^ of tissue. Finally, MECA-79-positive HEVs were calculated for each sample as counts per mm^2^ following the formula:

$${\mathrm{overall}}\,{\mathrm{HEVs}}\left[ {{\mathrm{counts}}/{\mathrm{mm}^2}} \right] = \frac{{{\sum} {{\mathrm{counts}}\,{\mathrm{intratumoural}}\,{\mathrm{and}}\,{\mathrm{peritumoural}}} }}{{{\mathrm{area}}\,{\mathrm{tumour}}\,{\mathrm{and}}\,{\mathrm{peritumoural}}}}$$

**Fig. 1:**
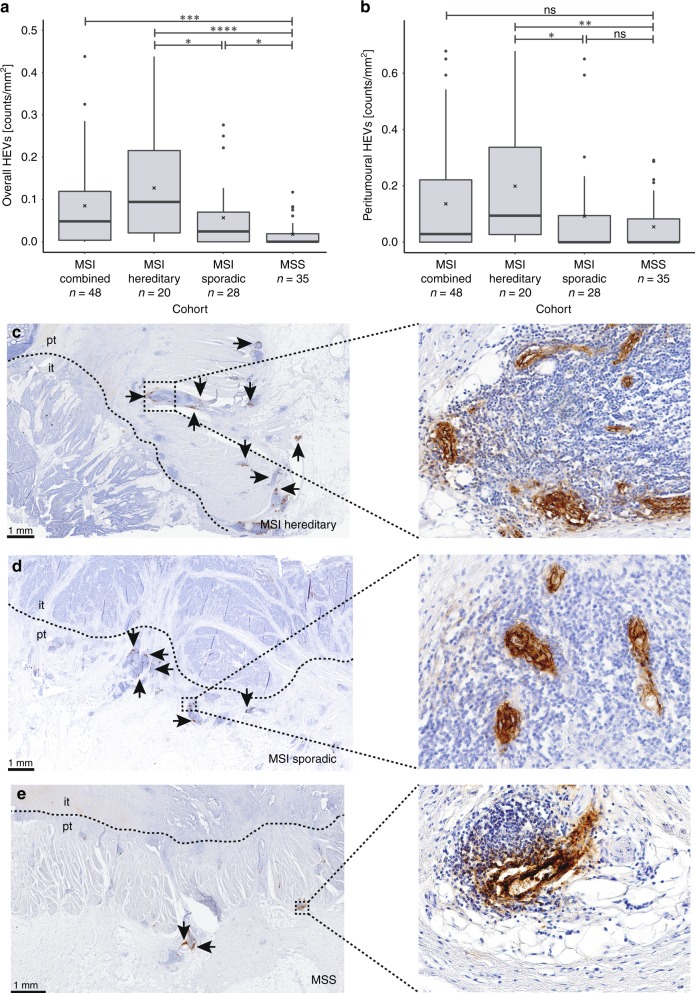
HEV density in sporadic MSI, hereditary MSI and MSS CRC samples. **a** Overall HEV density. MSI CRCs showed significantly elevated HEV densities as compared with MSS CRCs (*p* = 0.0002). Comparing MSI hereditary and sporadic MSI or MSS CRCs revealed a significantly elevated HEV density in hereditary MSI CRCs (*p* = 0.0255 and *p* = 3.9 × 10^–5^, respectively). MSI sporadic CRCs also showed significantly elevated HEV densities as compared with MSS CRCs (*p* = 0.0159). **b** Peritumoural HEV density. Considering only peritumoural HEVs, MSI CRCs with Lynch syndrome background revealed elevated HEV densities within this group, as compared with MSI sporadic and MSS (*p* = 0.010 and *p* = 0.0018, respectively). Black lines in boxes indicate the median value; cross indicates the mean value. Significance levels are depicted with asterisks (*p ≤ 0.05, **p ≤ 0.01, ***p ≤ 0.001, ****p ≤ 0.0001). **c** Representative image for HEV staining in MSI CRC of hereditary origin, **d** image for HEV staining in MSI CRC of sporadic origin and **e** presents HEV staining in MSS CRC. The boundary between the intratumoural (it) and peritumoural (pt) area is marked by a dashed line. Scale bar in **c**–**e** is 1 mm and HEVs are indicated by black arrows. All *p*-values were obtained from a nonparametric Wilcoxon rank-sum test

Intratumoural and peritumoural were calculated separately from each other (Fig. 1c–e, Supplementary Fig. S2). Counts in the peritumoural region were calculated as follows:

$${\mathrm{peritumoural}}\,{\mathrm{HEVs}}\left[ {{\mathrm{counts}}/{\mathrm{mm}^2}} \right]\\ = \frac{{{\sum} {{\mathrm{counts}}\,{\mathrm{peritumoural}}} }}{{{\mathrm{area}}\,{\mathrm{tumour}}\,{\mathrm{and}}\,{\mathrm{peritumoural}} - {\mathrm{area}}\,{\mathrm{tumour}}}}$$

and intratumoural HEV densities were calculated as

$${\mathrm{intratumoural}}\,{\mathrm{HEVs}}\left[ {{\mathrm{counts}}/{\mathrm{mm}^2}} \right] = \frac{{{\sum} {{\mathrm{counts}}\,{\mathrm{intratumoural}}} }}{{{\mathrm{area}}\,{\mathrm{tumour}}}}$$

Slides were scanned using a Hamamatsu NanoZoomer Digital Pathology system (Hamamatsu, Hamamatsu City, Japan) and analysed with NDP.view 2 software.

### Evaluation of PD-L1 expression

Samples were microscopically examined for PD-L1 expression and scored as negative, weak, medium or strong based on the percentage of staining-positive tissue area (0%, 1% to <10%, 10–40% and >40%, respectively). Scoring was performed independently by two observes, and observers were blinded during the scoring process.

### Evaluation of intratumoural CD3-positive T-cell density

Quantification of CD3 was performed as previously described.^19^ In digitised images of IHC staining, four random 0.25-mm^2^ squares were drawn and positive cells in the tumour region were counted, giving mean values per 0.25 mm^2^. Lymph follicles and germinal centres were identified on the basis of their morphological appearance and positive staining for CD20, and the counts were normalised per mm^2^ of the tissue.

### Preparation of age-independent cohorts

Samples from the MSI sporadic group were age-matched with samples from the hereditary MSI and MSS cohorts. Overall, 20 matches for each cohort could be performed. Age independency was assessed in a Welch two-sample t test (Supplementary Fig. S3b).

### *B2M*-mutation analysis

Mutation status of *B2M* was determined by Sanger sequencing as described previously.^8^ Briefly, PCR amplification of *B2M* exons 1 and 2 was performed using primer sequences: Exon 1 For—GGCATTCCTGAAGCTGACA, Exon 1 Rev— AGAGCGGGAGAGGAAGGAC, Exon 2a For—TTTCCCGATATTCCTCAGGTA, Exon 2a Rev—AATTCAGTGTAGTACAAGAG and Exon 2b For—TGTCTTTCAGCAAGGACTGG, Exon 2b Rev—CAAAGTCACATGGTTCACACG. After purification of the obtained PCR products (QIAquick PCR Purification Kit), the sequencing reaction was performed using the BigDye^®^ Terminator v1.1 Cycle Sequencing Kit (Thermo Fisher Scientific, Wilmington, DE, USA). Precipitated products were dissolved in 12 µl of HiDi Formamide (Thermo Fisher Scientific, Wilmington, DE, USA), sequenced on ABI 3130*xl* Genetic Analyzer and evaluated using Sequencing Analysis Software (Applied Biosystems).

### Statistical calculations

If not specified otherwise, *p*-values were calculated using the nonparametric Wilcoxon rank-sum test. For data following *t*-distribution, *p*-values were calculated using Welch two-sample *t* test, and for discrete values, a chi-square test was performed. As a measure of significance, *p* ≤ 0.05 was applied. All statistical analyses were performed in RStudio^20^ (version 1.1.453).

## Results

### HEV density in MSI and MSS colorectal cancers

We first aimed to analyse whether the density of HEVs in tumours is related to the MSI phenotype. By comparing 48 MSI against 35 MSS CRCs, we found a striking difference in the HEV density, with MSI CRCs having significantly higher HEV density (median MSI = 0.049 vs median MSS = 0.000 counts/mm^2^, *p* = 0.0002, Fig. 1). HEVs were preferentially located within the peritumoural region (43/54, 79.6% of HEV-positive CRCs) and less frequently found within the intratumoural area (22/54, 40.7% of HEV-positive CRCs); some tumours presented with both intra- and peritumoural HEVs (Fig. 1c–e, Supplementary Fig. S2). Intra- and peritumoural did not differ regarding morphology.

Our MSI cohort comprises sporadic and hereditary MSI CRCs. Therefore, we next analysed the density of HEVs in sporadic (*n* = 28) and hereditary (*n* = 20) MSI CRCs separately. The analysis revealed a significantly elevated overall HEV density (intra- and peritumoural) in MSI hereditary CRCs in comparison with their sporadic counterparts (median hereditary = 0.094 vs median sporadic = 0.025 counts/mm^2^, *p* = 0.0255, Fig. 1a). There was also a significant increase in the density of peritumoural HEVs: hereditary MSI CRCs presented with significantly elevated HEV densities as compared with sporadic counterparts (median = 0.136 vs median = 0.000 counts/mm^2^, *p* = 0.0104, Fig. 1b). Sporadic MSI CRC samples showed a significantly elevated HEV density (*p* = 0.0159, Fig. 1a), compared with MSS CRC (median MSI = 0.025 vs median MSS = 0.000 counts/mm^2^), when intra- and peritumoural HEVs were calculated together.

### Correlation of HEV density with clinical parameters

**Fig. 2:**
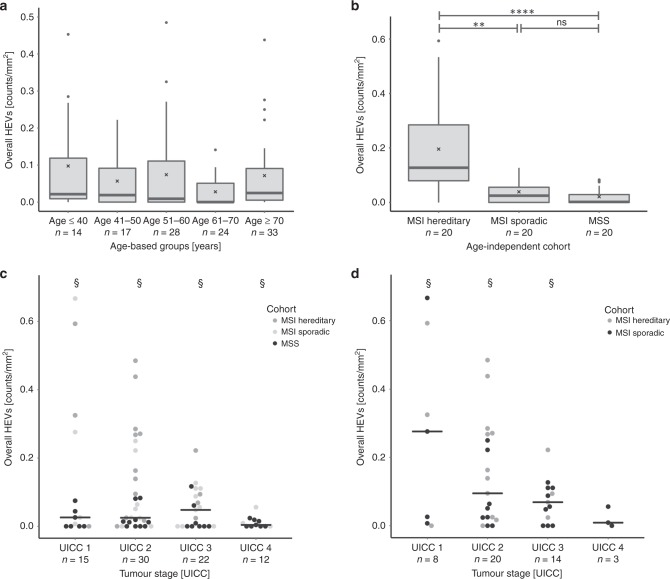
Correlation of HEV densities with clinical parameters. **a** HEV densities in tumours from patients diagnosed with CRC of any genetic background at different ages. **b** HEV density in tumours from age-matched groups of patients with hereditary MSI, sporadic MSI and MSS CRCs. MSI CRCs with Lynch syndrome background presented with significantly elevated HEV densities, as compared with MSI sporadic and MSS CRCs (*p* = 0.002 and *p* = 8.9 × 10^–6^). **c**, **d** HEV densities in relation to tumour stages **c** sporadic, hereditary MSI and MSS CRCs with different UICC stages were compared for their HEV densities; no significant differences were found. **d** MSI CRCs with different UICC stages were compared for their HEV densities; also, here, no significant differences in HEV densities were observed. The black line indicates the median value; cross indicates the mean value. Significance levels are depicted with asterisks (**—*p* ≤ 0.01 and ****—*p* ≤ 0.0001). All *p*-values were obtained from a nonparametric Wilcoxon rank-sum test. § represents outliers: UICC 1 group, *n* = 1, value = 2.44 counts/mm^2^; UICC 2 group, *n* = 1, value = 2.067 counts/mm^2^; UICC 3 group, *n* = 1, value = 1.889 counts/mm^2^; UICC four group, *n* = 1, value = 1.587 counts/mm^2^

Unlike sporadic cancer patients, Lynch syndrome patients are diagnosed with cancer at a younger age, due to genetic predisposition.^21,22^ We therefore asked whether the strong correlation of high HEV density with hereditary MSI tumour phenotype was a function of the younger age of Lynch syndrome patients, or possibly, whether this phenomenon was related to older age, due to long-term exposure of the immune system of Lynch syndrome patients to FSP *neo*antigens.^23–25^ First, we analysed the age distribution in our cohorts: as expected, age at CRC diagnosis differed significantly between three patient groups: while the mean age in the hereditary MSI CRC group was 52.7 years, the mean age of sporadic MSI CRC patients was 70.3 years, and the mean age of MSS CRC patients was 58.9 (Supplementary Fig. S3a). However, when we analysed the HEV density solely in relation to patients’ age at diagnosis, remarkably, we did not find any differences (Fig. 2a, Supplementary Fig. S3c). Similarly, no correlation was detectable between HEV density and patients’ age at diagnosis, upon dissecting the three different patient groups (Supplementary Fig. S3d–f).

As a next step, we compiled three age-matched groups with hereditary MSI, sporadic MSI and MSS CRC patients (Supplementary Fig. S3b). Comparison of HEV densities between these age-independent patient groups confirmed the significantly elevated HEV density in hereditary MSI CRC (median = 0.1275 counts/mm^2^) when compared with sporadic MSI (median = 0.0255 counts/mm^2^) and MSS (median = 0.002 counts/mm^2^) CRCs (*p* = 0.002 and *p* = 8.9 × 10^−6^, respectively, Fig. 2b).

Furthermore, we analysed the correlation of HEV densities with tumour stage. The distribution of HEV densities among different tumour stages suggested potentially higher HEV counts in tumours with a lower stage; however, statistical significance could not be reached, most likely due to limited sample size for each tumour stage (Fig. 2c, d).

### Correlation of HEV density with immune infiltration and immune evasion markers

**Fig. 3:**
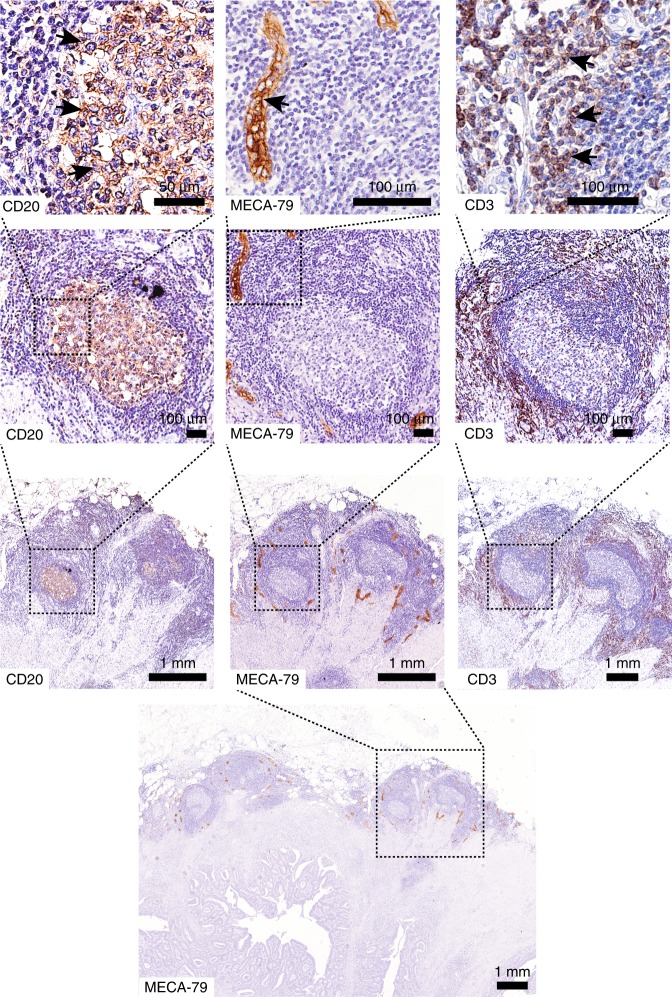
Relation of HEVs and immune infiltration. Staining of the same tumour sample for CD20 (left), MECA-79 (middle) and CD3 (right) markers. CD20-positive cells clearly marked the germinal centre of the lymph follicle, whereas CD3-positive cells were co-localised with HEVs. Arrows indicate the examples of positive staining. Scale bars top to bottom CD20: 50 µm, 100 µm, 1 mm; MECA-79: 100 µm, 100 µm, 1 mm, 1 mm; CD3: 100 µm, 100 µm, 1 mm

**Fig. 4:**
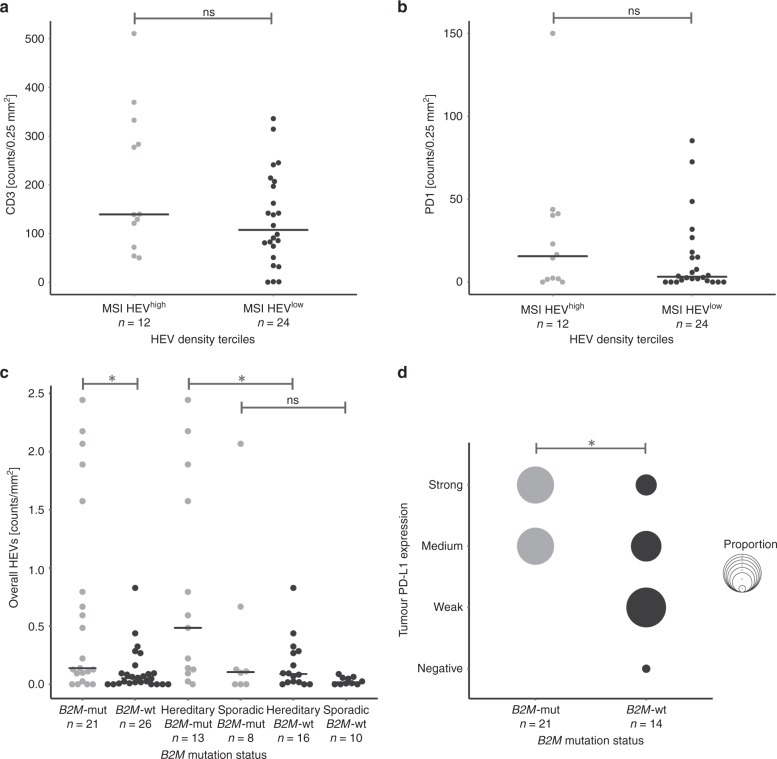
Association of HEV densities with immune response and immune evasion markers in MSI CRCs. **a** Immune infiltration detected with  CD3 marker compared in MSI tumours with high HEV densities (highest tercile) vs MSI CRCs with lower HEV densities (two lower terciles) reveals a tendency towards higher infiltration in HEV^high^ CRCs, although not reaching significance (*p* = 0.1637, Wilcoxon rank-sum test). **b** PD1-positive T lymphocytes in MSI CRCs are slightly higher in tumours with high HEV densities, as compared with those with lower HEV densities, but significance was not reached (*p* = 0.3361, Wilcoxon rank-sum test). **c** HEV densities in *B2M*-mutant (grey) and *B2M*-wild-type (black) MSI CRCs. HEV densities in *B2M*-mutant CRCs were significantly higher than in their *B2M*-wild-type counterparts (*p* = 0.0116, Wilcoxon rank-sum test). Upon dissecting MSI CRCs into hereditary and sporadic subgroups, *B2M*-mutant hereditary CRCs showed significantly elevated HEV densities, as compared with their *B2M*-wild-type counterparts (*p* = 0.0237) and **d** intratumoural PD-L1 expression was significantly higher in *B2M*-mutant MSI CRCs, as compared with their *B2M*-wild-type counterparts (*p* = 0.0161, Pearson’s chi-squared test). The black line indicates the median value. Significance levels are depicted with asterisks (*—*p* ≤ 0.05)

To investigate the relation between HEV density and immune infiltration, we first analysed the presence of CD3- and CD20-positive immune cells in the vicinity of HEVs. CD3-positive T cells were found to be localised directly around HEVs, whereas CD20-positive cells marked the germinal centres of the lymph follicles associated with the HEVs (Fig. 3, Supplementary Figs. S4 and S5). Next, intratumoural CD3-positive T-cell density was assessed in a subgroup of MECA-79-stained MSI CRCs. Although not reaching significance (p = 0.1637), a trend towards a higher CD3-positive cell infiltration in HEV^high^ tercile was observed, as compared with two terciles presenting with lower HEV densities (Fig. 4a). Furthermore, PD1-positive intratumoural T-lymphocyte infiltration was assessed. HEV^high^ MSI CRCs presented with higher PD1-positive T-cell infiltration, as compared with HEV^low^ counterparts. However, a significant difference was not observed (p = 0.3361, Fig. 4b). We also analysed the density of lymph follicles and germinal centres in MSI hereditary, MSI sporadic and MSS cohorts and correlated it with HEV densities. As expected, lymph follicle densities were significantly elevated in hereditary and sporadic MSI tumours compared with MSS tumours. Densities of lymph follicles with a germinal centre were also elevated in hereditary MSI tumours compared with MSS tumours. No significant difference in lymph follicle and germinal centre densities was observed between MSI hereditary and MSI sporadic tumours. Importantly, a significant correlation between HEV and lymph follicle densities, as well as between HEV and lymph follicle with germinal centre densities was observed (Supplementary Fig. S5 and S6c–f).

Mutations of the *B2M* gene have been previously reported in 30% of MSI CRCs, with a higher proportion of *B2M* mutations in Lynch syndrome-associated compared with sporadic MSI CRCs.^8^ We therefore analysed the HEV densities separately in *B2M*-mutant (*n* = 21) and *B2M*-wild-type (*n* = 26) MSI CRCs. Here, *B2M*-mutant tumours showed higher HEV densities compared with *B2M*-wild type tumours (median 0.139 vs 0.052 counts/mm^2^*, p* = 0.0116, Fig. 4c). We then analysed *B2M*-mutant hereditary MSI CRCs separately from sporadic ones and compared each of the groups with their *B2M*-wild-type counterparts. Among hereditary tumours, *B2M*-mutant MSI CRCs (*n* = 13) presented with significantly higher HEV density compared with *B2M*-wild-type (*n* = 16) tumours (median 0.485 vs 0.089 counts/mm^2^, p = 0.0237, Fig. 4c).

In addition to their high HEV density, *B2M*-mutant (*n* = 12) tumours also presented with stronger PD-L1 expression (*p* = 0.0161, Pearson’s chi-squared test) in comparison with their *B2M*-wild-type (*n* = 14) counterparts (Fig. 4d).

## Discussion

We observed an increased HEV density in MSI compared with MSS CRCs. To the best of our knowledge, this is the first study showing a relation between HEV density and MSI. A previous study addressing this question did not find a correlation of HEV densities with MSI status,^17^ most likely due to the limited number of only four MSI CRC samples analysed. The increased density of HEVs adjacent to MSI CRCs suggests that HEVs may contribute to the pronounced immune infiltration typically observed in MSI CRCs, by attracting circulating lymphocytes from the bloodstream directly to the tumour site.^4,5,26^

The increased density of HEVs in MSI tumours could be due to the high antigenic load and immunogenicity of MSI CRCs.^2^ However, the induction of HEV neogenesis in tumours is poorly understood. HEV neogenesis has been previously shown to occur under chronic inflammatory conditions,^14,27^ such as the Crohn’s-like reaction frequently observed in MSI tumours.^3,4^ One possible inducer of HEV neogenesis could be inflammatory cytokines, such as lymphotoxins. Lymphotoxin-α has been shown to have a role in HEV neogenesis. Furthermore, the lymphotoxin-αβ complex expressed by dendritic cells has been implicated in HEV neogenesis in mouse models and in human breast cancer cells.^15,28^ Recent findings describing HEV neogenesis upon tissue infiltration with activated T cells suggested a role for HEV in tumour destruction.^29,30^ A strong local immune response dependent on the recruitment of circulating lymphocytes by HEV could therefore be important for defeating MSI cancers. Studies analysing the induction of HEV neogenesis in MSI cancers are warranted, in order to identify potential targets for immune therapeutic approaches.

In line with previous publications, HEVs were in general more frequently found in peritumoural areas than intratumourally.^17,31^ Recognition by the MECA-79 antibody of a glycoantigen structure also present in a subset of CRCs (about 40%)^15,17,32–34^ resulted in positive staining of the tumour epithelium doppelte Beschreibung des subsets. However, this staining was clearly not indicative of HEVs and therefore was not accounted for by the observers (Supplementary Fig. S1a, b).

The comparison of HEV densities between sporadic and hereditary MSI CRCs revealed a significant difference, with higher HEV densities in hereditary MSI CRC. As sporadic MSI CRC patients are commonly of older age than hereditary MSI CRC patients,^21,22^ we suspected that the observed differences could be caused by the difference in the average age of patients from these two groups. However, age alone did not have a measurable influence on HEV densities. To exclude a possible influence of age on the observed differences, we compiled age-matched groups of sporadic and hereditary MSI and MSS CRC: the comparison of HEV counts in age-matched groups again indicated substantially higher HEV counts in the hereditary MSI group. Hence, these data suggest a fundamental difference in the anti-tumoural immune response between hereditary and sporadic MSI cancers, as even after age-matching, the observed differences remained significant. Our study supports previous observations of an elevated immune cell infiltration in hereditary compared with sporadic MSI colorectal cancers (reviewed by Shia et al.,^35,36^ Supplementary Fig. S6a, b). Furthermore, it suggests enhanced lymphocyte trafficking towards manifest tumours in Lynch syndrome individuals compared with sporadic MSI CRC patients and for the first time evaluates the contribution of HEVs to the immune microenvironment of different molecular CRC types, particularly delineating fundamental differences between immune response in hereditary and sporadic MSI CRC patients. Moreover, observation of a stronger immune infiltration in MSI hereditary endometrial tumours compared with sporadic ones,^37^ suggests that the difference between sporadic and hereditary MSI tumours may not be restricted to CRCs, but applies to a broad spectrum of Lynch syndrome-associated tumours. As expected, we also observed enhanced lymph follicle counts in MSI vs MSS CRC. However, differentiation between hereditary and sporadic MSI CRCs based on the lymph follicle counts alone was not statistically significant (Supplementary Fig. S6c, d). It is plausible to speculate that, in contrast to lymph follicle counts, which are less informative taken alone, HEVs reflect more the functional status of the immune milieu in a tumour.

Lynch syndrome mutation carriers have circulating T cells, specifically recognising MSI-induced *neo*antigens, which can be detected in peripheral blood even before cancer manifestation.^23^ Such immune responses are most likely triggered by MMR-deficient crypt foci harbouring mutations that give rise to FSP *neo*antigens.^24,25^ Previous studies have shown the relevance of immune cell recruitment from the bloodstream via HEVs into the site of inflammation for the successful immune response.^29,38–41^ In Lynch syndrome patients, HEVs may facilitate the recruitment of FSP-specific T cells, including recently primed cytotoxic CD8 T cells^42^ or naive T cells, which get activated on-site, from the blood to colon tissue affected by MMR-deficient lesions. This possibly contributes to their elimination by the local immune surveillance^43^ and may explain the limited penetrance of Lynch syndrome, with only 50% life-time CRC risk, despite the presence of thousands of MMR-deficient crypt foci in Lynch syndrome mutation carriers.^24^

**Fig. 5:**
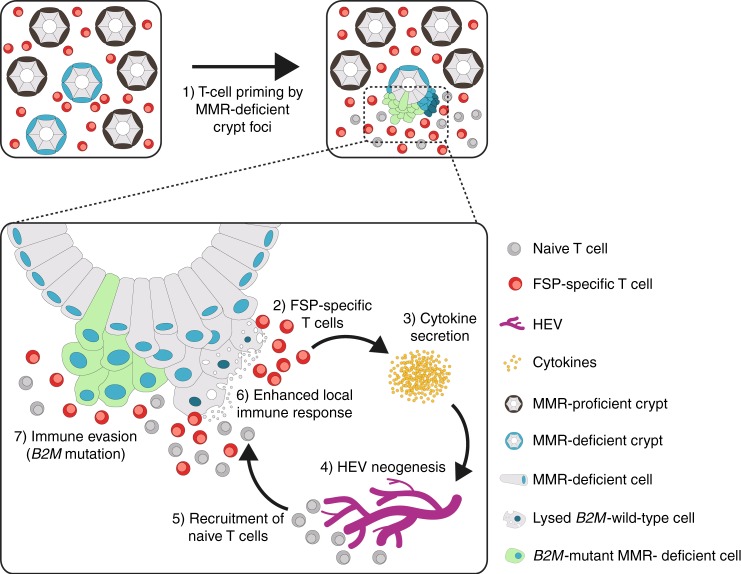
Graphical summary. Model of auto-immunisation loop in Lynch syndrome. In Lynch syndrome, the host immune system can be primed against FSP *neo*antigens by precursor lesions, MMR-deficient crypts, existing in the normal colonic mucosa long before tumour manifestation. Recurrent exposure of the immune cells to FSP *neo*antigens expressed in MMR-deficient crypts leads to the generation of an FSP-specific T-cell pool. When a tumour arises, FSP-specific T cells can promptly recognise the FSP *neo*antigens expressed on the tumour cells and release cytokines, which could induce neogenesis of HEVs. New HEVs, in turn, can recruit more naive T cells, which would undergo maturation through exposure to the *neo*antigens into FSP-specific T cells. Newly formed FSP-specific T cells release more cytokines, potentially leading to generation of more HEVs. This self-amplifying loop keeps MSI tumour cells under constant immune pressure and promotes selection and outgrowth of cell clones lacking antigen expression via MHC class I via acquisition of *B2M* mutations, enabling immune escape. Thereby, HEVs can contribute to immunoediting of MSI tumour cells.

It has been shown that MMR-deficient crypt foci are present in normal colonic mucosa of Lynch syndrome patients, but not in sporadic MSI CRC patients, with cancers arising from right-sided *BRAF*-mutant serrated lesions.^24,44,45^ A history of completely different precursor lesions in sporadic and hereditary MSI CRCs could be an explanation for a weaker immune activation in sporadic MSI CRCs compared with hereditary ones. Thus, it is conceivable that the lifelong autoimmunisation that takes place in the colonic mucosa of Lynch syndrome mutation carriers, but presumably not in sporadic MSI CRC patients, is responsible for the activation of the local immune microenvironment triggering HEV neogenesis via immune-activation signalling. The newly formed HEVs in turn recruit more lymphocytes, which may again induce HEV neogenesis, thereby constantly feeding the auto-immunisation loop. Such an auto-immunisation loop suggested to take place in Lynch syndrome-associated CRCs can be well integrated in the previously suggested self-amplifying loop between activated T cells and HEV neogenesis (Fig. 5), ultimately leading to possible elimination of precancerous or even cancerous lesions.^29^

We found significantly higher HEV densities in tumours with a *B2M* mutation in comparison with *B2M*-wild-type MSI CRCs. This association was observed even within the hereditary MSI group, which presented with the highest HEV counts among MSI CRCs. Previously, *B2M* mutations in MSI CRCs have been shown to correlate with a low number of FOXP3-positive T cells in the normal mucosa adjacent to tumour tissue, as well as with a high number of PD1-positive activated T cells in the tumour tissue, suggesting the role of active immune microenvironment in the induction of immune evasion.^19,46^ High HEV densities in *B2M*-mutant tumours corroborate these previous findings and imply that under strong immunoselective pressure created by immune cells recruited via HEVs, tumour cells which have lost MHC class I-associated antigen expression gain growth advantage, according to the immunoediting concept,^12^ thus revealing a major role of HEVs in enhancing the immunoselective pressure on highly immunogenic cancers. Taken together with the high HEV counts in Lynch syndrome CRCs, our findings point towards a longer process of immunoediting in Lynch syndrome CRCs, possibly due to pre-existing MMR-deficient crypts, explaining the higher proportion of *B2M* mutations in the tumours from Lynch syndrome patients compared with the sporadic ones.^8^

Taking into account the strong correlation of *B2M* mutations with better prognosis and low risk of distant metastasis in MSI CRCs,^8,47^ most likely explainable by lack of platelet binding essential for metastasis,^48^ HEV density may represent another prognostic marker for survival of patients with MSI CRC, particularly in Lynch syndrome scenario. In fact, a high density of HEVs may also contribute to local immune surveillance and the limited metastatic potential of MSI cancers.^4^ Studies analysing the functional impact of HEVs on metastasis formation are warranted.

In addition, HEVs could also potentially be of predictive value for treatment response towards immune checkpoint blockers (ICB). Previously, *B2M* mutations have been suggested to induce acquired resistance to ICB therapy.^49^ On the contrary, a recent study by Middha et al. demonstrated that patients with *B2M*-mutant CRCs can still achieve clinical benefit from ICB.^50^ Our study did not address the predictive value of HEV densities for ICB therapy. However, *B2M-*mutant tumours in our study not only had elevated HEV densities, but also higher levels of PD-L1 expression, suggesting secretion of IFN-gamma as a result of a prolonged immune interaction, which in turn favoured the acquisition of *B2M* mutation, leading to loss of antigen presentation via MHC class I and immune escape. The implications of this finding with regard to the predictive value of HEVs for immunotherapy in MSI cancers should be addressed by future studies.

It is important to note that our study has an observational design, and in order to draw functional conclusions regarding HEVs and their role in anticancer immunity, further studies using suitable animal models are required.

In conclusion, our data provide evidence that the elevated HEV densities reflect the active immune milieu in MSI colorectal tumours. HEVs may support recruitment of FSP-specific T cells to MSI cancer tissues, thereby contributing to a strong immune- selective pressure that favours the outgrowth of cell clones, which acquired immune evasion mechanisms. Our findings underline the impact of immune surveillance in cancer and corroborate the concept of immunoediting in tumour evolution.

## Supplementary information

Supplementary Data

## Data Availability

All presented data are available at the Department of Applied Tumor Biology and can be provided upon request.
