# Supplementary material for: High endothelial venules are associated with microsatellite instability, hereditary background and immune evasion in colorectal cancer
Source: Br J Cancer. 2019 Jul 30;121(5):395–404. doi: 10.1038/s41416-019-0514-6 (PMC6738093; doi:10.1038/s41416-019-0514-6)
Supplement: Supplementary file 1 — Supplementary Data [file 41416_2019_514_MOESM1_ESM.docx]

**SUPPLEMENTARY FIGURES**

**Supplementary Figure S1**


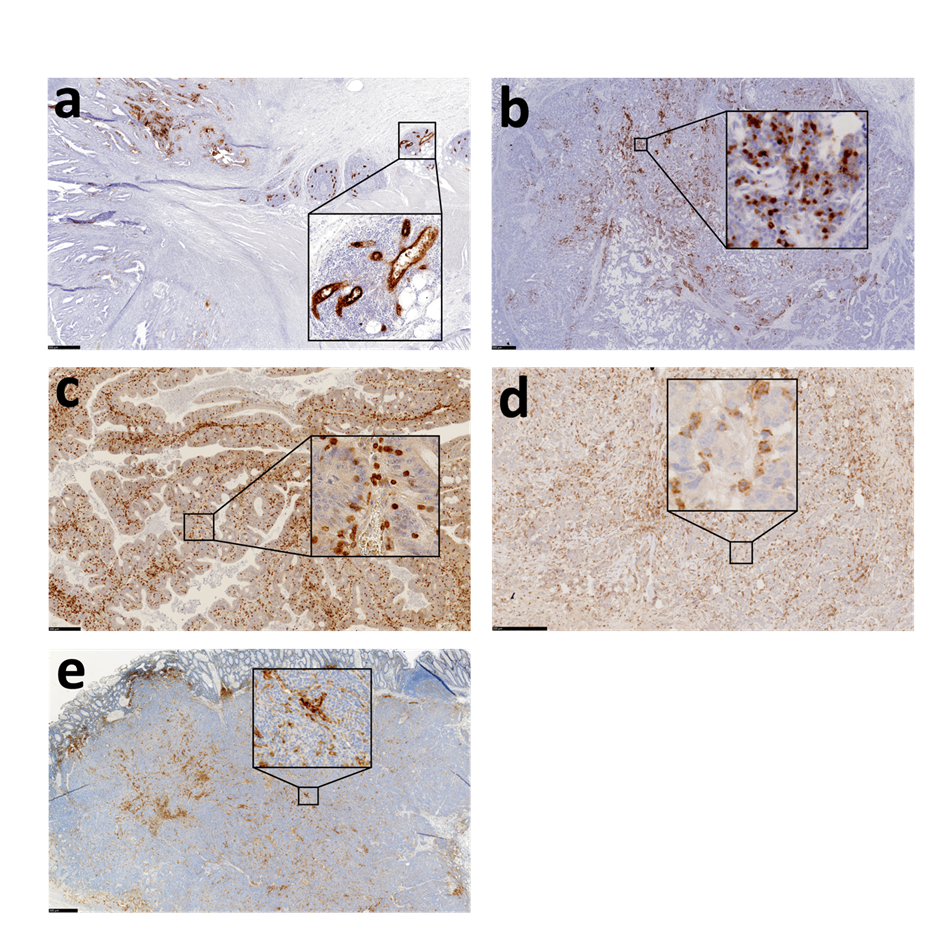


**Supplementary Figure S1. Detection of HEVs using MECA-79 antibody. a) MECA-79 positive staining of HEVs (scale bar=500 µm). The majority of detected HEVs were located in the peritumoral area, with only few samples presenting with HEVs within the normal or tumor epithelium. b) Epithelial MECA-79 expression by tumor cells (scale bar=500 µm). 40 out of 83 tumors presented with MECA-79 positivity of the epithelial cells, ranging between 5% and 70%. However, this positive staining was not indicative of HEVs, but due to specificity of MECA-79 antibody to 6-sulpho sialyl-Lewis^x^ antigen, indicated the expression of 6-sulpho sialyl-Lewis^x^ antigen by tumor epithelial cells, also reported in previously published literature ^32^ c) CD3-positive T cells in intratumoral region of MSI CRC. d) PD1-positive T cell infiltrating the tumor region. e) PD-L1 expression by tumor cells. Scale bar in a, b and e=500µm and in c and d=200µm.**

**Supplementary Figure S2**

**
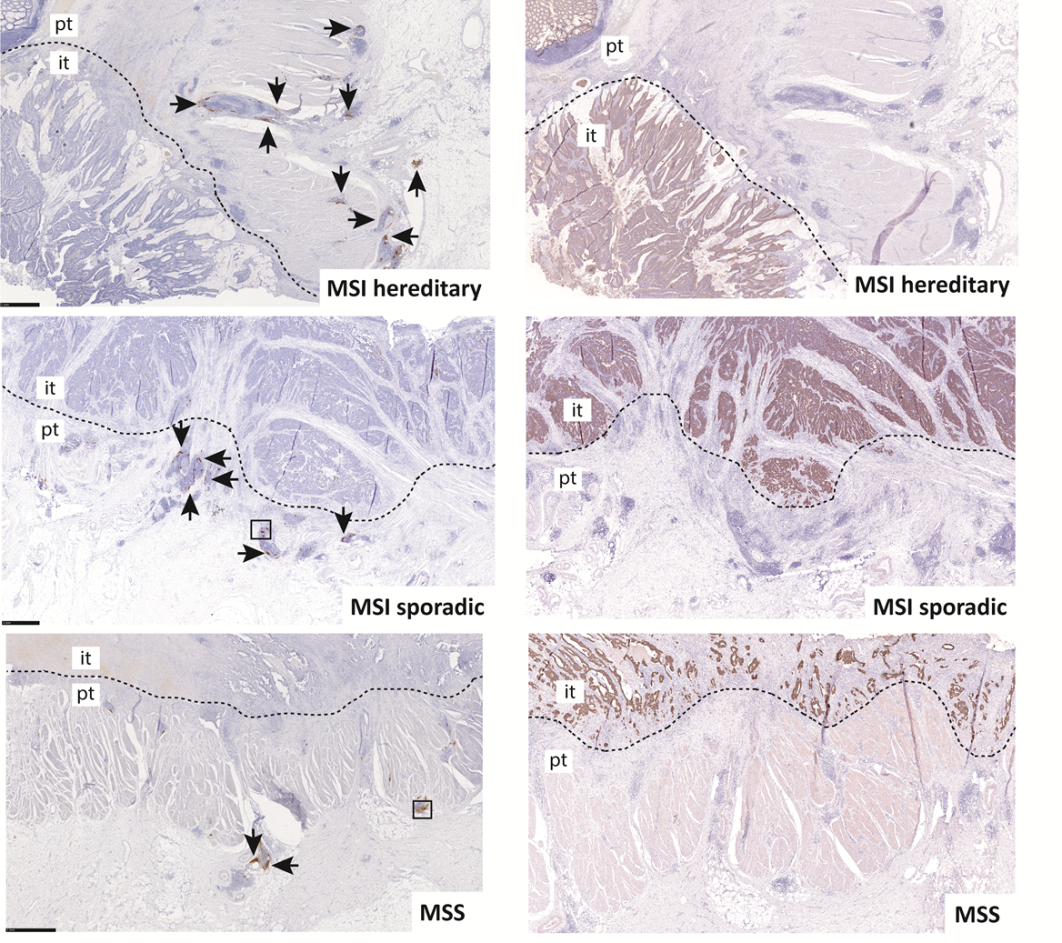
**

**Supplementary Figure S2. HEV (left) and cytokeratin (right) stainings of FFPE tumor sections for the discrimination of intratumoral and peritumoral regions. The cytokeratin staining marks the epithelial cells helping to distinguish between intra- and peritumoral HEVs.**

**Supplementary Figure S3**


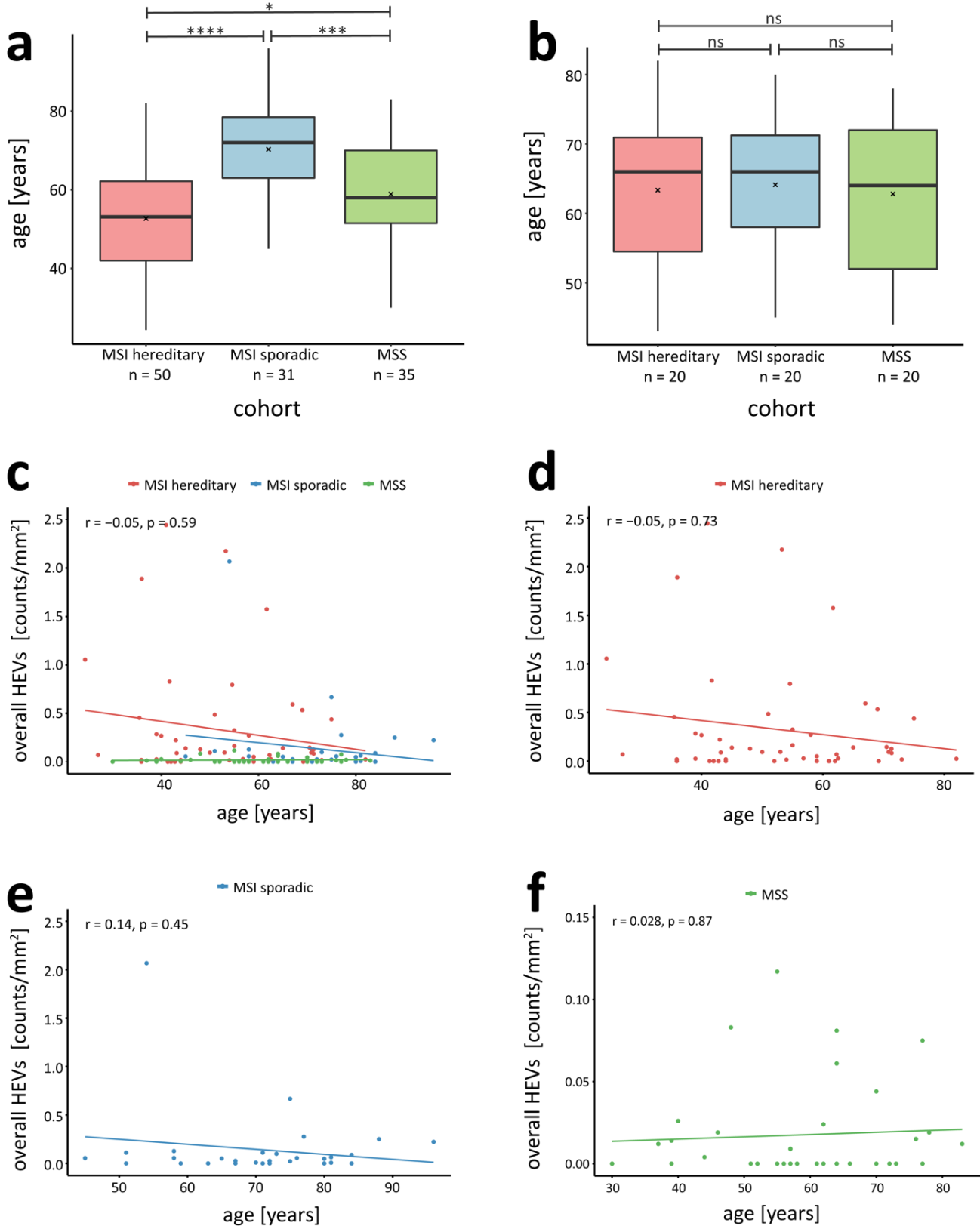


**Supplementary Figure S3. Age distribution and preparation of age-independent MSI and MSS cohorts. a) Original age distribution within MSI hereditary, MSI sporadic and MSS patients. Ages differed significantly between all three groups (hereditary vs sporadic p=3.5x10^-8^, hereditary vs MSS p =0.038 and sporadic vs MSS p =0.0005, Welch Two Sample t-test). b) age-independent cohorts prepared as described in Methods, p-values were obtained from a Welch Two Sample t-test. c-f) Spearman correlation between patients' ages and HEV density reveals no direct relation between age and presence of HEVs.**

**Supplementary Figure S4**

**
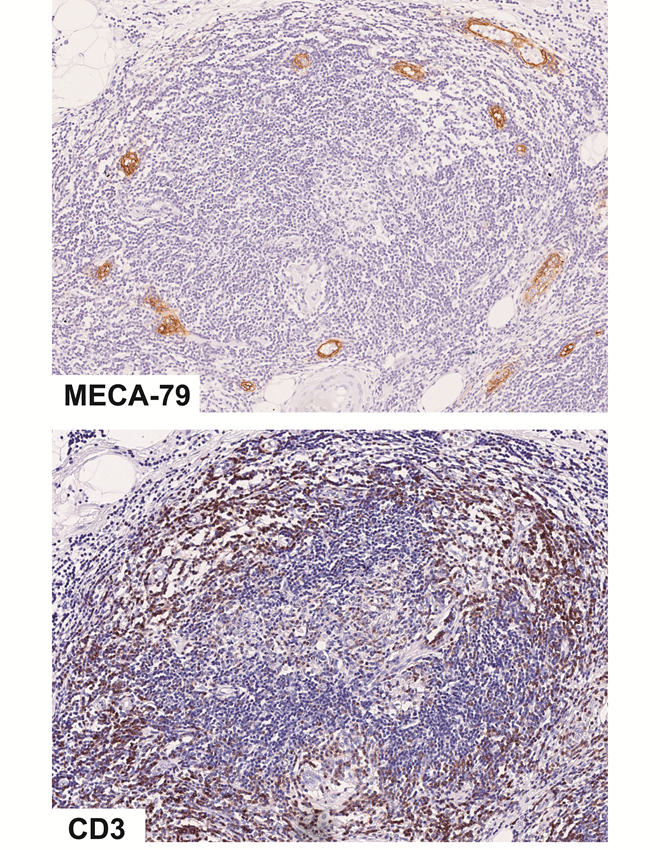
**

**Supplementary Figure S4. HEV (upper panel) and CD3 (lower panel) stainings of the same section.**

**Supplementary Figure S5**

**
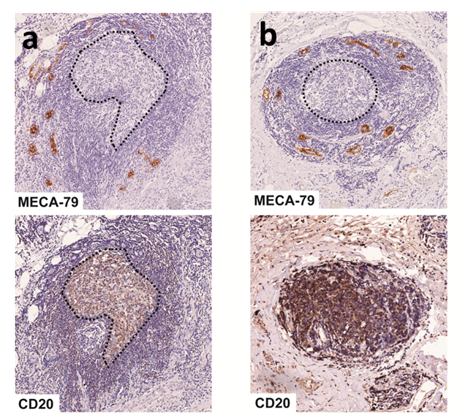
**

**Supplementary Figure S5. Representative MECA-79 (upper panel) and CD20 (lower panel) stainings of the same tumor sections. CD20 staining marks the germinal centers of the lymph follicles (a and b). Lymph follicles without recognizable germinal centers did not show diffuse positive signals for CD20 staining.**

Supplementary Figure S6





Supplementary Figure S6. CD3- and PD1-positive T cell infiltration in MSI CRCs, lymph follicle and germinal center counts in MSI and MSS CRCs. a) Comparison between MSI hereditary and MSI sporadic group regarding intratumoral CD3-positive T cell infiltration revealed significantly elevated infiltration with CD3-positive T cells (p=0.009) in MSI hereditary group. b) Infiltration with activated (PD1-positive) T cells was significantly higher in MSI CRCs of hereditary background as compared to their sporadic counterparts (p=0.0123). c) Lymph follicles were counted and normalized per mm^2^ as described for HEVs. Combined MSI cohort as well as MSI hereditary and sporadic CRCs considered solely revealed significantly higher lymph follicle densities as compared to MSS CRCs (p=4.5*10^-6^, p=0.0002 and p=10*10^-5^, respectively). d) Lymph follicle density correlates with HEV density (r=0.66, p=3.3*10^-11^, Spearman correlation). e) MSI CRCs with Lynch Syndrome background as well as combined MSI cohort presented with significantly elevated numbers of germinal centers compared to MSS CRCs (p=0.0062 and p=0.0311). f) Density of germinal centers correlates with HEV density (r=0.54, p=3.5*10^-7^). If not specified otherwise, all p-values were obtained from a non-parametric Wilcoxon Rank Sum Test.
